# Defence Responses Associated with Elicitor-Induced, Cultivar-Associated Resistance to Latania Scale in Kiwifruit

**DOI:** 10.3390/plants11010010

**Published:** 2021-12-21

**Authors:** Kirstin Wurms, Annette Ah Chee, Kate Stannard, Rachelle Anderson, Dwayne Jensen, Janine Cooney, Duncan Hedderley

**Affiliations:** 1 The New Zealand Institute for Plant and Food Research Limited (Plant & Food Research), Private Bag 3230, Waikato Mail Centre, Hamilton 3240, New Zealand; Annette.AhChee@plantandfood.co.nz (A.A.C.); Dwayne.Jensen@plantandfood.co.nz (D.J.); janine.cooney@plantandfood.co.nz (J.C.); 2 Plant & Food Research, 412 No. 1 Road, RD2, Te Puke 3182, New Zealand; Kate.Stannard@plantandfood.co.nz (K.S.); Rachelle.Anderson@plantandfood.co.nz (R.A.); 3 Plant & Food Research, Private Bag 11600, Palmerston North 4442, New Zealand; Duncan.Hedderley@plantandfood.co.nz

**Keywords:** Actigard, cross-talk, induced resistance, host defence response, pathogenesis-related (PR) protein, salicylic acid

## Abstract

Latania scale insect is a pest of global significance affecting kiwifruit. The sessile insect (life stage: settled crawler—mature adult) is covered with a waxy cap that protects it from topical pesticides, so increasingly, a selection of resistant cultivars and application of elicitors are being used in pest control. Thus far, the application of a salicylic acid (SA) phytohormone pathway elicitor, acibenzolar-S-methyl (ASM), has been shown to reduce insect development (as indicated by cap size) on one kiwifruit cultivar (‘Hayward’). To investigate how cultivar-associated resistance is affected by the ability to respond to different elicitors, we measured phytohormones (by LCMS) and gene expression (by qPCR and NanoString) on latania scale-tolerant ‘Hort16A’ and susceptible ‘Hayward’ kiwifruit over two seasons. Potted plants in the presence/absence of settled latania scales were treated with ASM (0.2 g/L) or methyl jasmonate (MeJA, 0.05% *v*/*v*), representing elicitors of the SA and JA signalling pathways, respectively. ‘Hort16A’ cultivar resistance to latania scale was associated with elevated expression of SA and SA-related defence genes (*PR1* and two *PR2* family genes) in the ASM treatment. MeJA treatments did not significantly affect insect development in ‘Hayward’ (latania scale did not survive on ‘Hort16A’) and did not correlate with phytohormone and gene expression measurements in either cultivar. ‘Hayward’ had greater concentrations than ‘Hort16A’ of inert storage forms of both SA and JA across all treatments. This information contributes to the selection of tolerant cultivars and the effective use of elicitors for control of latania scale in kiwifruit.

## 1. Introduction

Latania scale (*Hemiberlesia lataniae* Signoret), a sap-sucking insect, is one of two significant armoured scale insect pests on kiwifruit (*Actinidia chinensis*), which is the most important horticultural export from New Zealand [1] and a key crop in Italy, China, and Chile [2]. Latania is the predominant scale on New Zealand kiwifruit [3], where it is mainly important as a market access pest [4]. Armoured scale insects are uniparental and reproduce parthenogenetically. The mobile dispersal stage (crawler) that emerges from the egg beneath the adult’s scale cap survives for up to one day before settling permanently on the host, spinning a protective cap over itself (which is firmly attached to the plant host), and remains sessile for the remainder of its life cycle. The cap grows as the insect body underneath increases in size, with the insect going through two moults before achieving maturity, approximately 10 weeks after settlement on kiwifruit [5]. Latania scale is highly polyphagous, and its ability to survive on more than 78 families of host plants, together with its worldwide distribution, make it an invasive pest of global significance [6]. The insect tends to be resistant to pesticides with contact-only activity since apart from the crawler stage, it spends all of its lifecycle sessile and covered with a hard waxy protective cap. Consequently, chemical control can be particularly challenging, and host resistance and biological control represent the most promising control alternatives [7]. Despite this, there are numerous published papers on host resistance to chewing insects, but relatively fewer studies on resistance to sap-sucking insects [8,9].

Up until 2011, with the discovery of *Pseudomonas syringae* pv. *actinidiae* (Psa) in New Zealand, nearly all New Zealand’s kiwifruit exports came from two cultivars: green-fleshed *Actinidia chinensis* var. *deliciosa* ‘Hayward’ and yellow-fleshed *Actinidia chinensis* var. *chinensis* ‘Hort16A’ [5]. Pest and disease resistance were not used as selection criteria for developing new cultivars, such as ‘Hort16A’ in the early 1990s, but subsequently, it was found that ‘Hort16A’ was very tolerant to the latania scale, whilst ‘Hayward’ was susceptible [5]. In addition, histological investigations demonstrated that ‘Hort16A’ demonstrated a hypersensitive-like response to latania scale feeding, including the formation of a wound periderm, along with lignification, suberisation, phenolic accumulation, and plant cell necrosis around and beneath the insect. This wound periderm developed over the course of 3–5 weeks, stopping the stylet from reaching unmodified parenchyma tissue and, thus, preventing the settled insect from growing and reaching maturity, with death usually occurring in the first and second instars [10]. The results suggest the involvement of both physical and chemical defence responses in ‘Hort16A’ resistance. To further elucidate defence pathways, a transcriptome study was initiated that looked at the expression of 17,512 genes associated with latania scale infestation of ‘Hort16A’ kiwifruit [3]. In common with plant defence responses to other sap-sucking insects [11], latania scale infestation resulted in marked up-regulation of genes associated with the salicylic acid (SA) pathway, as well as genes involved in the biosynthesis of secondary metabolites, such as anthocyanins, chalcones, and flavanols. In contrast, genes from the jasmonic acid (JA) pathway, which tend to be antagonistic to the SA pathway [12], were down-regulated [3]. Furthermore, exogenous application of an SA-mimic, acibenzolar-S-methyl (ASM), caused a significant 23% reduction in the growth of latania scale (as measured by its cap size) on the normally susceptible ‘Hayward’ plants [3].

However, several questions remain unanswered with the research carried out to date. Results indicated that ‘Hort16A’ resistance to latania scale is mediated primarily via the SA defence pathway [3], but no measurements have been made on the phytohormones themselves. Furthermore, there has not been any comparison of the response in tolerant versus susceptible kiwifruit cultivars or any investigation of the effect of eliciting the antagonistic jasmonic acid (JA) pathway. Thus, it is essential to understand the effects of hormonal crosstalk because we do not wish the use of a particular elicitor to inhibit one problem to inadvertently increase susceptibility to another. Resistant cultivars and elicitors are being increasingly used in pest and disease control, so to address these gaps in our knowledge and to test our hypotheses, this research aimed to:

1. Use phytohormone and gene expression measurements to investigate whether cultivar-specific resistance is determined by responsiveness to different elicitors (including the pest itself, as well as elicitors of the antagonistic SA versus JA pathways).
2. Examine the effects of hormonal crosstalk on the host–pest interaction.

We hypothesise that ‘Hort16A’ is tolerant to the latania scale because it is more responsive than ‘Hayward’ to SA-pathway elicitors, and that use of a JA pathway elicitor should facilitate latania growth and survival (assuming typical antagonistic SA–JA pathway crosstalk).

This information contributes to the selection of tolerant cultivars and the effective use of elicitors for control of latania scale in kiwifruit.

## 2. Results

### 2.1. MeJA Elicitor Application Did Not Affect Latania Scale Growth

‘Hort16A’ is naturally highly tolerant to latania scale; thus, none of the scale insects survived beyond two weeks on the ‘Hort16A’ plants, irrespective of treatment. Over the course of 10 weeks, the latania scale developed naturally on both MeJA-treated and control ‘Hayward’ plants, with no significant treatment differences in insect growth and development (as indicated by cap size) (*p* = 0.868).

### 2.2. Changes in Phytohormones and Gene Expression in Elicitor-Induced, Cultivar-Associated Resistance to Latania Scale

#### 2.2.1. Phytohormone Measurements

**Figure 1:**
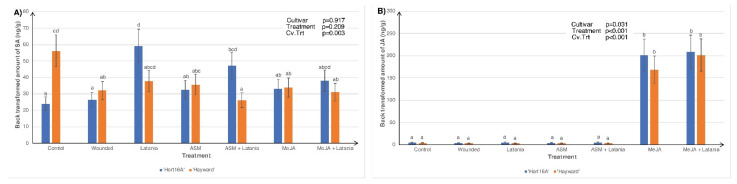
Phytohormone concentrations of (**A**) salicylic acid (SA) and (**B**) jasmonic acid (JA) in ‘Hort16A’ and ‘Hayward’ kiwifruit leaf tissue in 2016. Leaves were left unsprayed or were treated with 0.2 g/L Actigard™ (ASM) to induce the SA pathway or with 0.05% *v*/*v* methyl jasmonate (MeJA) to elicit the JA pathway. Latania scale crawlers (approximately 200/leaf) were applied seven days later, or leaf wounding (to imitate scale feeding) was achieved with a fine needle. Control plants were left untreated. Seven days after scale application, leaves were sampled for phytohormone measurements. There were five replicate plants per treatment. The Y-axis concentration scale is different for each phytohormone. Probability values from ANOVA are shown in the top right-hand corner of each bar chart, and each chart plots the cultivar × treatment interaction (Cv.Trt). Error bars give the standard errors, and different lettering (a, b, c, d) indicates statistically significant treatment differences, as shown by Fisher’s Least Significant Difference (LSD), *p* ≤ 0.05.

**Figure 2:**
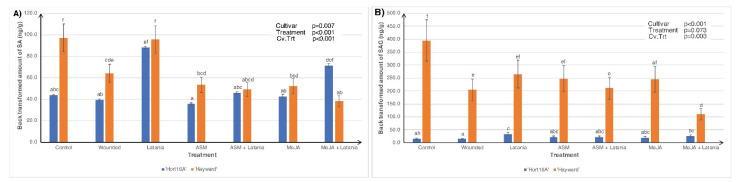
Phytohormone concentrations measured in ‘Hort16A’ and ‘Hayward’ kiwifruit leaf tissue, in 2018 of salicylic acid (SA) in (**A**); and salicylic acid glucoside (SAG)—an inert storage form of SA, in (**B**). Leaves were left unsprayed or were treated with 0.2 g/L Actigard™ (ASM) to induce the SA pathway or with 0.05% *v*/*v* methyl jasmonate (MeJA) to elicit the jasmonic acid (JA) pathway. Latania scale crawlers (approximately 200/leaf) were applied seven days later, or leaf wounding (to imitate scale feeding) was achieved with a fine needle. Control plants were left untreated. Although leaves were sampled for phytohormone measurements both two and seven days post scale infestation (psi), only the two-day psi results were significant and presented here. There were five replicate plants per treatment. The Y-axis concentration scale is different for each phytohormone. *p*-values from ANOVA are shown in the top right-hand corner of each bar chart, and each chart plots the cultivar × treatment interaction (Cv.Trt). Error bars give the standard errors, and different lettering (a, b, c, d, e, f) indicates statistically significant treatment differences, as shown by LSD, *p* ≤ 0.05.

**Figure 3:**
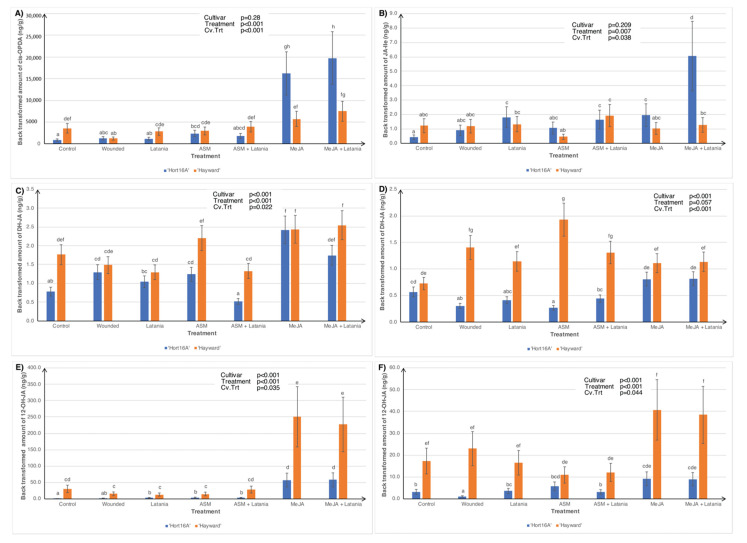
Phytohormone concentrations in ‘Hort16A’ and ‘Hayward’ kiwifruit leaf tissue, in 2018, of cis-12-oxyphytodienoic acid (cis-OPDA)—a JA precursor, at two days post latania scale infestation (psi), in (**A**); jasmonic acid isoleucine (JA-Ile)—the most bioactive form of JA, at two days psi, in (**B**); dihydrojasmonic acid (DH-JA)—a less active form of JA, at two days psi, in (**C**); DH-JA, at seven days psi, in (**D**); 12-hydroxy jasmonic acid (12-OH-JA)—an inert storage form of JA, at two days psi, in (**E**); and 12-OH-JA, at seven days psi, in (**F**). Treatments are described in full in Figure 2. Although leaves were sampled for both two and seven days psi for phytohormone measurements, only the statistically significant cultivar × treatment interactions (Cv.Trt) are presented. There were five replicate plants per treatment. The Y-axis concentration scale is different for each phytohormone. *p*-values from ANOVA are shown in the top right-hand corner of each bar chart. Error bars give the standard errors, and different lettering (a–h) indicates statistically significant treatment differences, as shown by LSD, *p* ≤ 0.05.

Measurements in 2016 were only made at seven days post scale infestation (psi) because there was insufficient scale settlement on day two, and therefore the plants required re-seeding, and the only phytohormones measured were SA, JA, and ABA (Figure 1). However, measurements in 2018 were made for both two and seven days psi and were extended to include active and inactive forms of SA and JA (Figure 2 and Figure 3).

In 2016, constitutive SA levels were higher in ‘Hayward’, but the introduction of the latania scale markedly induced SA in the ‘Hort16A’ cultivar (Figure 1A). MeJA application significantly increased JA in both cultivars (Figure 1B). The interaction between treatment and cultivar (Cv.Trt) had no significant effect on ABA levels; hence the data are not presented.

In 2018, similar to the 2016 results, constitutive SA levels were higher in ‘Hayward’, but the introduction of latania scale insect markedly induced SA in the ‘Hort16A’ cultivar two days post scale infestation (psi) (Figure 2A). However, patterns of SA expression were similar at both two and seven days psi, the Cv.Trt interaction was not significant at seven days psi, so the data are not presented. Concentrations of the inert storage form of SA (SAG) were significantly higher in ‘Hayward’ than in ‘Hort16A’ irrespective of the treatment and collection time, but the Cv.Trt interaction was only significant at two days, with SAG being lowest in the MeJA + Latania treatment in ‘Hayward’ (Figure 2B).

Contrary to the 2016 results, none of the treatments in 2018, including MeJA, had any significant effect on JA levels in either cultivar; hence the data are not shown. However, levels of the precursor of JA (cis-12-oxyphytodienoic acid (cis-OPDA)) were significantly higher in leaves from the MeJA treatments than those from all other treatments, especially in ‘Hort16A’ at two days psi (Figure 3A). The same trend was seen with the most bioactive form of JA (jasmonic acid isoleucine (JA-Ile)) in the MeJA + Latania treatment in ‘Hort16A’ at two days psi only (Figure 3B). In contrast, the less active (dihydrojasmonic acid (DH-JA)) and inert (12-hydroxy jasmonic acid (12-OH-JA)) forms of JA were present in significantly higher concentrations in ‘Hayward’ than in ‘Hort16A’, especially in the ASM treatments for DH-JA at both two and seven days psi (Figure 3C,D) and in the MeJA treatments for 12-OH-JA at both two and seven days psi (Figure 3E,F). Concentrations of 12-OH-JA were greater at two days than at seven days post scale application (Figure 3E,F). In common with the 2016 results, Cv.Trt interactions were not significant at either psi time for the 2018 ABA data; thus, they are not presented.

#### 2.2.2. Gene Expression

**Figure 4:**
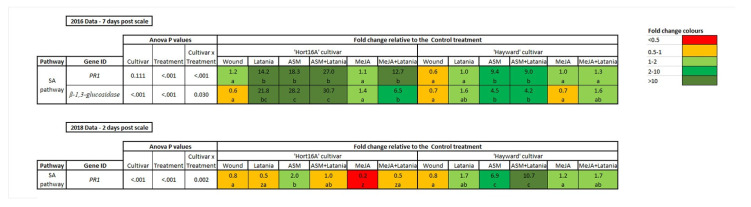
Relative defence gene expression as measured by quantitative polymerase chain reaction (qPCR) in ‘Hort16A’ and ‘Hayward’ kiwifruit leaf tissue in 2016 and 2018. Leaves were left unsprayed or were treated with 0.2 g/L Actigard™ (ASM) or with 0.05% *v*/*v* methyl jasmonate (MeJA) to elicit the salicylic acid (SA) and jasmonic acid pathways, respectively. Seven days later, latania scale crawlers (approximately 200/leaf) were applied, or leaf wounding (to imitate scale feeding) was carried out using a fine needle. Control plants were left untreated. Leaf sampling for gene expression analysis took place seven days post scale infestation (psi) in 2016 and at two and seven days in 2018. There were five replicate plants per treatment. Fold change data relative to the control treatment are presented, where orange/red colours represent a decrease in gene expression, whilst green shades indicate up-regulation relative to the control. Fold changes in the order of 0.5–2 are not considered biologically significant [13,14,15]. Statistical analysis necessitated a log_2_ transformation, but data are presented as back-transformed means ± standard errors. *p*-values are from ANOVA, and only data with a significant Cultivar × Treatment interaction are presented. Different lettering indicates statistically significant treatment differences relative to the control treatment (designated the letter a), as shown by LSD (*p* ≤ 0.05), with the letter z indicating significant down-regulation.

In 2016 gene expression was measured by qPCR (seven days post infestation data only), but in 2018 data from both two and seven days after latania scale infestation were measured by qPCR and the newer NanoString technology. Figure 4 shows the qPCR measurements, in both 2016 and 2018, of genes of interest (GoI) for which the Cv.Trt interaction was statistically significant, and these genes were only from the SA pathway. Genes without any statistically significant Cv.Trt interaction are not presented. In 2016, the ASM and latania scale treatments showed statistically significant up-regulation of *PR1* and β-1,3-glucosidase in both cultivars, but up-regulation was up to 14-fold higher in ‘Hort16A’ than in ‘Hayward’ (Figure 4). In 2018, a significant Cv.Trt interaction was only observed for *PR1* at two days psi. The basal levels of PR1 in ‘Hort16A’ were much higher in ‘Hayward’, so only the ASM treatment caused a significant increase in ‘Hort16A’ relative to the control, whereas the MeJA treatment resulted in a significant decrease in *PR1* expression. The ASM treatments caused a significant increase in *PR1* expression in ‘Hayward’ relative to the control treatment.

**Figure 5:**
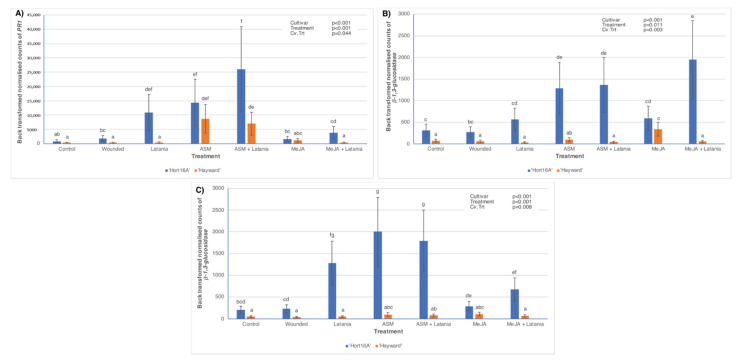
Defence gene expression measured by PlexSet^®^ NanoString in ‘Hort16A’ and ‘Hayward’ kiwifruit leaf tissue, in 2018, of salicylic acid (SA) pathway-related genes: *PR1*, at seven days post scale infestation (psi) in (**A**); and *β-1,3-glucosidase*, at two days and seven days psi in (**B**) and (**C**) respectively. Treatments are described in full in Figure 2. Although leaves were sampled from both two and seven days psi for gene expression measurements, only the statistically significant cultivar × treatment interactions (Cv.Trt) are presented. There were five replicate plants per treatment. The Y-axis represents physical counts of the number of molecules expressed. Statistical analysis necessitated a log_2_ transformation, but data are presented as back-transformed means ± standard errors. The Y-axis scale is different for each gene. *p*-values from ANOVA are shown in the top right-hand corner of each bar chart. Different lettering (a–g) indicates statistically significant treatment differences, as shown by LSD, *p* ≤ 0.05.

In 2018, gene expression was also measured by NanoString, which included the most successful genes as determined by qPCR, as well as a wider range of genes associated with the SA pathway, the JA pathway, and the secondary metabolite phenylpropanoid pathway. Figure 5 shows NanoString expression in 2018 of SA-pathway genes that also demonstrated significant expression in qPCR (shown in Figure 4). Result trends in Figure 5 were similar to those observed with qPCR, i.e., latania scale and ASM treatments were most effective at up-regulating the expression of *PR1* and *β-1,3-glucosidase*, and increases were much greater in ‘Hort16A’ than in ‘Hayward’.

**Figure 6:**
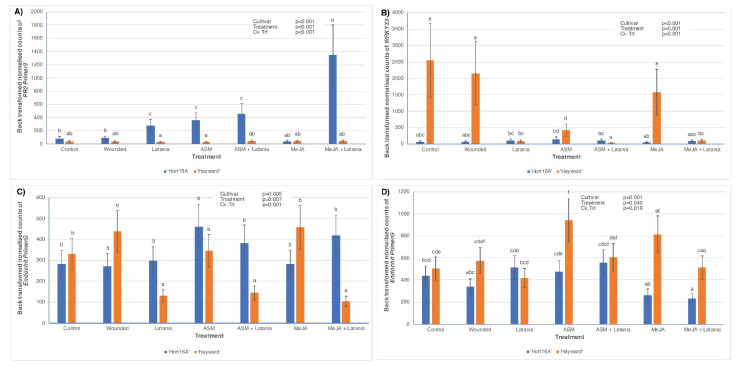
Defence gene expression measured by PlexSet^®^ NanoString in ‘Hort16A’ and ‘Hayward’ kiwifruit leaf tissue, in 2018, of salicylic acid (SA) pathway-related genes: *PR2 Primer3*, at two days post scale infestation (psi), in (**A**); *WRKY33*, at two days psi in (**B**), and *Endochitinase* (*Endochit PrimerG*), at two and seven days psi, in (**C**) and (**D**) respectively. Treatments are described in full in Figure 2. Although leaves were sampled from both two and seven days psi for gene expression measurements, only the statistically significant cultivar × treatment interactions (Cv.Trt) are presented. There were five replicate plants per treatment. The Y-axis represents physical counts of the number of molecules expressed. Statistical analysis necessitated a log_2_ transformation, but data are presented as back-transformed means ± standard errors. The Y-axis scale is different for each gene. *p*-values from ANOVA are shown in the top right-hand corner of each bar chart. Different lettering indicates statistically significant treatment differences, as shown by LSD, *p* ≤ 0.05.

Other SA pathway-related genes, which exhibited significant expression and were measured by NanoString in 2018, are shown in Figure 6. *PR2 Primer3* was only significantly induced in ‘Hort16A’ by the Latania treatments (especially MeJA + Latania) and ASM at two days psi (Figure 6A). At two days psi *WRKY33* was markedly expressed only in susceptible ‘Hayward’ and was highest in the Control, Wounded, and MeJA treatments, suggesting that it was repressed by latania scale infestation and ASM application (Figure 6B). The presence of the latania scale appeared to repress expression of *Endochit PrimerG* in ‘Hayward’ but not in ‘Hort16A’ at two days psi (Figure 6C). However, at seven days psi, the most marked treatment differences were observed in the ASM, MeJa, and MeJA + Latania treatments, where expression was higher in ‘Hayward’ than in ‘Hort16A’ (Figure 6D).

**Figure 7:**
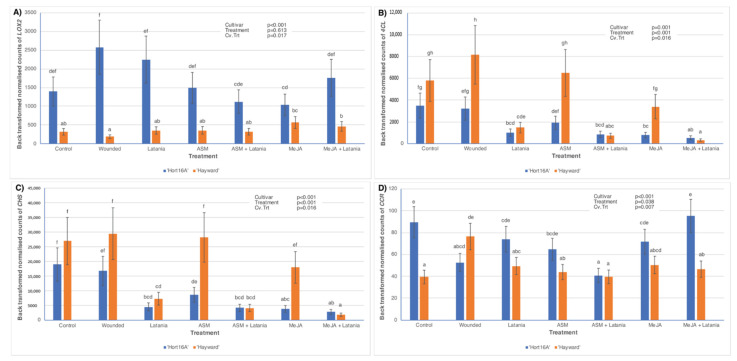
Defence gene expression measured by PlexSet^®^ NanoString in ‘Hort16A’ and ‘Hayward’ kiwifruit leaf tissue, in 2018, of jasmonic acid (JA) and phenylpropanoid pathway-related genes: *Lipoxygenase* (*LOX2*), at seven days post scale infestation (psi), in (**A**); *4-Coumarate coenzyme A ligase* (*4CL*), at two days psi, in (**B**); *Chalcone synthase* (*CHS*), at two days psi, in (**C**); and *Cinnamoyl-coenzyme A reductase* (*CCR*), at seven days psi, in (**D**). Treatments are described in full in Figure 2. Although leaves were sampled from both two and seven days psi for gene expression measurements, only the statistically significant cultivar × treatment interactions (Cv.Trt) are presented. There were five replicate plants per treatment. The Y-axis represents physical counts of the number of molecules expressed. Statistical analysis necessitated a log_2_ transformation, but data are presented as back-transformed means ± standard errors. The Y-axis scale is different for each gene. *p*-values from ANOVA are shown in the top right-hand corner of each bar chart. Different lettering indicates statistically significant treatment differences, as shown by LSD, *p* ≤ 0.05.

JA-pathway and secondary metabolite-pathway genes that exhibited a significant Cv.Trt interaction, and were measured by NanoString in 2018, are shown in Figure 7. At seven days psi, *LOX2* expression was consistently higher in ‘Hort16A’ than in the ‘Hayward’ cultivar, even in the Control, and there was no significant treatment effect in either cultivar (Figure 7A). Two genes from the phenylpropanoid pathway (PPP) that produces secondary metabolites, *4CL*, and *CHS*, showed similar trends in that expression at two days psi tended to be inhibited by latania scale in both cultivars (Figure 7B,C). CCR leves were much higher in the Control in ‘Hort16A’ than in ‘Hayward’, with significant down-regulation occurring in the ASM + Latania treatment for ‘Hort16A’ and the highest expression of CCR occurring in the Wounded treatment for ‘Hayward’ (Figure 7D).

## 3. Discussion

The phytohormone and gene expression data from both the 2016 and 2018 experiments on latania scale-tolerant ‘Hort16A’ and susceptible ‘Hayward’ kiwifruit plants support the conclusions that 1) ‘Hort16A’ cultivar resistance to latania scale is associated with greater elicitation of the SA pathway by latania scale infestation and ASM treatment than in ‘Hayward’, and 2) antagonistic crosstalk between the SA and JA pathways does not seem to occur in the kiwifruit–latania scale interaction, hence use of a JA pathway elicitor does not facilitate latania scale growth and survival. Latania scale infestation led to elevated expression of SA phytohormone, together with the up-regulation of SA-related defence genes (namely *PR1*, *β-1,3-glucosidase*, and *PR2 Primer3*) in the ASM and latania scale treatments in ‘Hort16A’. The same treatments resulted in suppression of another SA-related defence gene (*Endochit PrimerG*) in ‘Hayward’ and were associated with either reduced latania scale growth (as indicated by its cap size) on ‘Hayward’ [3] or complete mortality on ‘Hort16A’ (this study). In contrast, the MeJA treatment did not significantly affect the expression of JA-related genes in either cultivar. Furthermore, the MeJA treatments did not increase latania scale growth in ‘Hayward’ or survival on ‘Hort16A’. Whilst gene expression proved to be a more reliable marker of resistance than phytohormones, both active and inert forms of the phytohormones were measured in 2018, with results suggesting that cycling between different storage forms may play an important role in phytohormone regulation. ‘Hayward’ had greater concentrations than ‘Hort16A’ of the inert storage forms of both SA and JA across all treatments. In the MeJA treatments, in particular, the JA precursor (cis-OPDA) was elevated in ‘Hort16A‘, whilst the concentration of the inert form of JA (12-OH-JA) increased the most in the same treatments in ‘Hayward’. Phytohormone and gene measurements from the ABA pathway did not indicate any involvement in elicitor-induced, cultivar-associated resistance to the latania scale.

### 3.1. Cultivar Response to SA Pathway Elicitation

Although ‘Hayward’ had higher constitutive levels of SA (and SAG) than ‘Hort16A’, the increase in SA following latania scale infestation was more significant in ‘Hort16A’ than in ‘Hayward’ (when compared to their relative controls), which could be a trigger for subsequent enhanced SA-related gene expression in ‘Hort16A’. Although ASM is a SA analogue, it operates downstream of SA synthesis and was not expected to increase SA in either cultivar [16]. SA concentrations vary considerably between plant species [17,18], and comparing constitutive concentrations does not predict relative differences in gene expression or capacity to resist pests and diseases. Consequently, the more relevant comparison is to look at changes in SA concentrations following the introduction of the latania scale. 

Phytohormone measurements, in general, are less clear cut than the measurements of specific defence genes because phytohormones are involved in just about every aspect of a plant’s growth and response to its environment. In addition to antagonistic crosstalk between different pathways, phytohormones can be influenced by many factors, including tissue type, age, external abiotic and biotic factors, and circadian rhythms [19,20,21,22]. Moreover, the spatial and temporal regulation of hormone biosynthesis is highly complex [20,23], meaning that the timing and sampling strategies are critical. Using a pooled leaf sample for the phytohormone analysis was necessary because of limitations on the amount of scale-seeded tissue available but would have contributed to variability by diluting the direct effect of scale infestation with non-infested leaves. A more comprehensive sampling strategy with the greater temporal and spatial resolution is recommended for future research.

Out of a total of eleven SA pathway genes examined, the expression of three of these candidates (*PR1*, *β-1,3-glucosidase*, *PR2 Primer3*) has been shown to correlate positively with elicitor-induced resistance to the latania scale. Treatment with the SA pathway analogue, ASM, and latania scale infestation, led to increased expression of SA pathway-associated genes (*PR1*; and *β-1,3-glucosidase*—a member of the *PR2* family) in ‘Hort16A’ versus ‘Hayward’. These results were validated by carrying out trials in two different years and with two different methods of gene measurement. NanoString measurements also showed positive correlations between the ASM and latania treatments and ‘Hort16A’ resistance for another *PR2* SA-associated gene (*PR2 Primer3*). Genes from the *PR1* and *PR2* families are commonly used SA-pathway markers [24,25,26] that have proved to be consistently effective in SA-mediated kiwifruit defence response against various pests and pathogens [3,27,28,29,30,31,32]. Although the most stable reference genes used for the qPCR work in 2016 (*40s ribosomal protein* and *Actin*) were different from those used in 2018 (*GAPDH* and *PP2A*), the conclusions were validated by the NanoString measurement in 2018, where all the different references genes were used simultaneously for normalisation.

*PR1* plays a crucial role in the activation of systemic acquired resistance [33], and previous transcriptome analysis of the ‘Hort16A’ resistance response to latania scale infestation showed strong up-regulation of the specific *PR1* protein (Acc06864.1) used in the current study [3]. *PR1s* may inhibit insect growth via sequestration of sterols, which are essential for growth and as structural components of cell membranes [34,35]. This mode of action is especially effective against sterol auxotrophs, such as most insects, which cannot manufacture their own sterols and therefore need to obtain them from their environment, typically via metabolising phytosterols [35].

*The β-glucosidases* [E.C.3.2.1.21], which encompass the subfamily of *β-1,3-glucanases* (*PR2* proteins), are the enzymes that hydrolyse glycosides/oligosaccharides and are functionally vital to biological systems [36] because they convert inert storage forms of compounds, such as phytohormones and pesticidal secondary metabolites, into their corresponding active forms by removal of sugar residues [37]. Cycling between active and inert storage pools of metabolites allows for faster responses than would be possible by de novo synthesis, which often requires multistep biosynthetic pathways. Interestingly, in the 2018 trial, two key enzymes in the secondary metabolite phenylpropanoid pathway (*CHS, 4CL*) were suppressed in both cultivars across the latania treatments. A third enzyme (*CCR*) had significantly lower expression in the ASM + Latania treatment versus the Control in ‘Hort16A’, yet we know that phenolics, callose, and lignin accumulate as part of the ‘Hort16A’ hypersensitive response to latania scale infestation that leads to insect death [10]. Since it is unlikely that inactive family members have been selected from three different gene families, a more likely explanation is that the *β-glucosidases*/*PR2* proteins, which are more highly up-regulated in tolerant ‘Hort16A’ than susceptible ‘Hayward’, are activating inert storage pools of these secondary metabolites in ‘Hort16A’. In support of this hypothesis, glycosylation has shown to be a significant regulator of phenylpropanoid availability, biological activity, and plant stress responses [38,39,40], and deglycosylation of phenolic extracts from kiwifruit has been shown to greatly increase their antifungal activity [41].

Finally, two further SA-associated genes (*Endochit PrimerG*, *WRKY33*) were down-regulated by the ASM and latania scale treatments in the ‘Hayward’ cultivar. Expression of *Endochit PrimerG*, a member of the *PR3* gene family, was repressed in the presence of latania scale in susceptible ‘Hayward’ but not in tolerant ‘Hort16A’ at two days psi. *WRKY33* expression also appeared to be repressed in the latania scale and ASM treatments in ‘Hayward’ but not in ‘Hort16A’. It has been shown that *WRKY33* is induced by salicylate and binds to more than 1000 gene loci, many of which are involved in biotic and abiotic stress responses and is, therefore, thought to regulate crosstalk between the SA and JA pathways [42,43,44]. However, it is most commonly associated with enhanced resistance to necrotrophic pathogens via antagonistic crosstalk between the JA and SA pathways [44].

### 3.2. Cultivar Response to JA Pathway Elicitation

Defences induced by stylet-feeding arthropods can include both JA and SA defences [45,46], but JA pathway induction was not as strong as SA pathway induction in any of the treatments or either cultivar. Results suggest that cycling between active and inert storage pools may play an important role in phytohormone regulation [47,48]. Although the application of the JA pathway elicitor, MeJA, did result in induction of active forms of JA especially in ‘Hort16A’, it also resulted in greater accumulation of inert forms of JA in ‘Hayward’. This would also help explain why MeJA treatments did not facilitate latania scale growth on ‘Hayward’ because the application of this elicitor simply increased the pool of inert forms of JA in this cultivar.

Of the five JA-associated genes tested in this study, none appeared to be affected by elicitor-induced, cultivar-associated resistance. The JA-associated gene, *LOX2*, showed the only significant Cv.Trt interaction, but this related to higher expression of *LOX2* in latania scale-tolerant ‘Hort16A’ than in susceptible ‘Hayward’, irrespective of treatment. This may help explain why ‘Hort16A’ is also known to be more resistant than ‘Hayward’ to the necrotic pathogen, *Botrytis cinerea*, and to the chewing insect, brownheaded leafroller [49], both of which are thought to activate the JA pathway. The finding illustrates that the SA and JA pathways are not always directly antagonistic and sometimes have neutral or even synergistic interactions [50,51,52].

### 3.3. The Importance of Crosstalk

Of equal and sometimes greater importance than elicitation of hormonal pathways themselves is the interaction or feedback between these pathways, commonly referred to as “crosstalk” [12,53]. In most crosstalk models, the JA and ABA pathways are directly antagonistic to the SA pathway [54,55], although examples of neutral or even synergistic interactions have also been recorded [45,50,51,52]. If reciprocal SA–JA antagonism was the case for the kiwifruit–latania scale interaction, elicitation of the JA pathway might be expected to increase latania scale survival in tolerant ‘Hort16A’ and increase insect growth rate (as measured by its cap size) in susceptible ‘Hayward’. However, MeJA treatment had no effect on latania scale growth or survival and increased the level of inert forms of JA in the ‘Hayward’ versus ‘Hort16A’. Moreover, MeJA did not induce significant gene expression in any of the five JA-associated marker genes. Finally, *WRKY33* transcriptional regulation has been associated with up-regulation of JA-associated genes and concomitant down-regulation of SA pathway genes [44]. However, the suppression of *WRKY33* in the latania scale and ASM treatments in ‘Hayward ‘did not result in large increases in SA-associated gene expression in ‘Hayward’ relative to ‘Hort16A’. These observations are suggestive of a neutral interaction between the SA and JA pathways for the kiwifruit–latania scale interaction.

Measurements of the phytohormone ABA and two ABA-pathway genes (*ABA1* and *RD22*) did not show any significant Cv.Trt interactions. Down-regulation of *ABA1* [28] or no significant change in expression of *RD22* and *ABA1* [32] have been observed in other studies of the kiwifruit SA-mediated defence response to bacterial canker. However, differences between these results may be due to the differences in sampling times and a different pathogen. Whilst results from the current study do not support the hypothesis that the ABA and SA pathways are antagonistic, conclusions cannot be drawn from such a small sample size of ABA genes and active versus inert forms of the ABA phytohormone were not measured, hence further investigation is warranted.

### 3.4. Future Directions and Impact

To further extend this research in the future, phytohormones and gene expression will be measured in newer cultivars in commercial production, such as the grape-sized *Actinidia arguta* (latania scale-tolerant) and *Actinidia chinensis* var. *chinensis* ‘Zesy002′ (latania scale-susceptible). It will also be valuable to investigate more genes associated with regulating glycosylated storage pools of inert forms of phytohormones and secondary metabolites, such as *UDP-glucosyltransferase*, which performs the reverse role to *β-1,3-glucosidase*, in that it glycosylates moieties [56]. The research has identified interesting candidate SA-pathway genes that appear to play a role in elicitor-induced, cultivar-associated resistance to the latania scale. However, evidence to date is correlative only and does not establish whether their roles in resistance are direct or indirect. Consequently, the next stage of the project will be to test the functionality of these candidate genes, either through overexpression, silencing/knockouts, or gene editing.

Besides identifying candidate gene markers suitable for monitoring the latania scale-kiwifruit interaction, this research has also shown the value of studying active versus inert storage pools of defence phytohormones. Previous work has shown that ASM, an SA-pathway elicitor, can successfully reduce latania scale growth [3]. The current study expands this knowledge by showing that using a JA-pathway elicitor has no effect on latania scale growth, and there does not appear to be antagonistic crosstalk between the SA and JA pathways. This information contributes to the selection of tolerant cultivars and the effective use of elicitors for control of latania scale in kiwifruit.

## 4. Materials and Methods

### 4.1. Plants and Insects

Two experiments (in 2016 and 2018) were carried out on *Actinidia chinensis* var. *chinensis* ‘Hort16A’ (latania scale-tolerant) and *Actinidia chinensis* var. *deliciosa* ‘Hayward’ (latania scale-susceptible) potted plants. Tissue-cultured plantlets were obtained from Multiflora (Auckland, New Zealand) and were exflasked from an agar growth medium in sealed plastic tubs into 0.5-L planter bags filled to 2/3 with Daltons™ GB potting mix (Daltons, Matamata, New Zealand) and topped up with a 50:50 mix of potting mix and perlite. The plants were placed in a glasshouse (15–24 °C, 14 h day length) within high humidity tents with supplementary bottom heating for the first two weeks of growth. Plants were then transferred to a flood and drain table. In 2016, the plants were flooded twice daily for 10 min each, with plain water, and received a once-weekly foliar feed of 3.3 mL/L Yates Thrive^®^. In 2018 the plants were flooded only once daily, with a hydroponic nutrient solution (pH 6.2) containing calcium, iron, nitrates, sulphates, phosphates, and trace elements (PGO Horticulture Ltd., Tīrau, New Zealand). After six weeks of growth, when plants were about 30 cm tall with 3–6 fully expanded leaves, treatments were applied.

The wildtype latania scale (*Hemiberlesia lataniae* Signoret) population used for these trials was initially isolated in 2002 from unsprayed ‘Hayward’ vines at a Plant & Food Research (PFR) orchard (Te Puke, New Zealand), and colonies have been maintained since that time by rearing on mature fruit of butternut squash (*Cucurbita moschata*) at constant temperature (20 ± 2 °C) and humidity (65 ± 5%) [3]. Crawlers (the newly enclosed, mobile stage of the first instar) were seeded onto ‘Hort16A’ and ‘Hayward’.

### 4.2. Treatment Application and Sampling Procedure

Seven treatments were applied to both kiwifruit cultivars (Table 1). There were two sample collection times (two days and seven days after the latania scale application and mechanical wounding treatments were applied) made on separate plants, and there were five replicate plants per treatment/time combination, set up in a randomised block design.

For the wounding treatment, the sample leaf was punctured 20 times with a 1-mL BD Ultra-Fine™ needle. An artist’s paintbrush was used to seed 200+ crawlers onto the adaxial surface of the sample leaf on each plant.

Acibenzolar-S-methyl (ASM, used at 0.2 g/L), commercially known as Actigard™, was used as a SA pathway elicitor [3], and methyl jasmonate (MeJA, used at 0.05 *v*/*v* + 0.025% (*v*/*v*) DuWett^®^) as a JA pathway elicitor [57]. All elicitor spray treatments were applied on day one of the trials, using a hand-held 1-L spray bottle, with a separate bottle used for each treatment. Spraying took place inside a plastic tent, washed down with water between treatments. Each plant was sprayed separately, and both sides of every leaf were sprayed to run off. MeJA treatments (which can result in volatiles) were applied last, and the plants were kept separate from the others (in identical conditions) for 24 h to avoid cross-treatment contamination.

A single healthy, fully grown sample leaf (S) was selected at the mid-point of the height of each plant for the scale infestation treatments and the wounding treatment. The relative position of the sample leaf on each plant was standardised because leaves nearest the base of the plant are the oldest, whilst those closest to the apex are the most immature. In 2016, scale settlement was extremely low at the two-day post infestation collection point, so a decision was made at day two to seed additional scale onto the sample leaves of the plants set aside for the seven-day collection.

Leaf tissue samples for phytohormone and gene expression analyses were collected two and seven days after the scale crawlers, and mechanical wounding treatments were applied. In 2016, three leaf discs (approximately 100 mg of tissue) were collected using an 18-mm diameter cork borer from each sample leaf (S) and were pooled together for gene expression analysis, but this sample size was increased to nine discs from the sample leaf in 2018. For phytohormone analysis, three leaf discs (c. 100 mg) were taken from each sample leaf (S), as well as three discs from the leaf immediately above (U) and below (L) the sample leaf and all nine discs were pooled together. Different sampling procedures had to be used for the phytohormone and gene analyses because of limits on the size of the total crawler population, such that there were only enough crawlers to place on the sample leaf, and the sample leaf could not provide enough material for both types of analysis. All samples were snap-frozen in liquid nitrogen and stored at −80 °C until processing for phytohormone and gene expression analyses.

### 4.3. Measurement of Latania Scale Growth/Survival in the Methyl Jasmonate (MeJA) Treatment

After completing the 2018 experiment, the untreated control plants of ‘Hayward’ and ‘Hort16A’ were re-potted. For each kiwifruit cultivar, there were two spray treatments (water + 0.025% *v*/*v* DuWett^®^ or methyl jasmonate 0.05% *v*/*v* + 0.025% *v*/*v* DuWett), with ten replicate plants per treatment. (Measurement of latania scale growth/survival with ASM had already been determined previously, so was not repeated [3]). The plants were set up in a randomised block design in the glasshouse and spaced so that the leaves were not touching. Seven days after the first spray application, a minimum of 100 scale crawlers were applied using a fine paintbrush to the stem midway up each plant. The stem was used rather than the leaves because a bioassay has shown that latania scale will reach maturity (defined as a cap size of ≥ 1 mm, when the scale can produce eggs) after 10 weeks at 20 °C, and leaves are not a reliable substrate to use over this length of time [5]. After seven days, five settled crawlers per plant were tagged using a dot of nontoxic paint placed adjacent to the scale, with insect #1 being closest to the apex of the plant and insect #5 closest to the base of the plant. Following the first treatment application, a further three spray applications of MeJA and water were made at three weekly intervals. Photographs were taken at 4, 6, 8 and 10 weeks after the first treatment application, and growth of the scale was quantified by measuring the cap size of tagged individuals in the photographs (using J Fiji software, version 1.51w). Latania maturity was achieved in 10 weeks, and all tagged scales were checked at this time for mortality and evidence of egg production by flipping the caps at the end and examining them under a dissecting microscope (Wild Heerbrugg M38). At this time, live insects under the caps were yellow and turgid in appearance, whilst dead insect bodies were brown and desiccated.

### 4.4. Phytohormone Measurements

Phytohormones were measured by liquid chromatography/mass spectrophotometry (LC/MS) and quantitated using stable isotopically labelled standards. In 2016 phytohormone measurements only included salicylic acid (SA), jasmonic acid (JA), and abscisic acid (ABA), with leaf samples prepared for analysis as fresh frozen ground tissue (100 mg fresh weight (FW)) and extracted according to Supplementary Protocol S1. In 2018 measurements were expanded to include conjugates of SA and JA, representing both bioactive and inert storage forms, with the methodology described in Bulley, et al. [58]. Leaf samples in 2018 (100 mg FW) were extracted using Supplementary Protocol S2 to improve the retention and recovery of more polar phytohormones, such as salicylic acid glucoside.

Formic acid (Riedel-de Haën) was purchased from Sigma Aldrich (Auckland, New Zealand); Optima LC/MS grade acetonitrile and trifluoroacetic acid (TFA) from Thermo Fisher Scientific (Auckland, New Zealand); and water was of Milli-Q grade. All other compounds used in LC/MS analysis are shown in Supplementary Table S1.

LC/MS measurements were performed on a 5500 QTrap triple quadrupole/linear ion trap (QqLIT) mass spectrometer equipped with a TurboIon-Spray™ interface (AB Sciex, ON, Canada) coupled to a Shimadzu Exion UHPLC (Kyoto, Japan). Plant hormones were separated on a Poroshell 120 SB-C18 2.7 μm 2.1 × 150 mm ID column (Agilent Technologies, CA, USA) maintained at 60 °C. Solvents were (A) water + 0.1% formic acid and (B) acetonitrile + 0.1% formic acid, and the flow rate was 400 μL^−1^. In the initial mobile phase, 2% B was held for 3 min before ramping linearly to 16% B at 3.5 min, then to 100% B at 7 min and holding at 100% B until 8 min before resetting to the original conditions. The injection size was 10 μL. Multiple reaction monitoring transitions used for plant hormone analysis are shown in Supplementary Table S2. Other operating parameters were as follows: dwell time, 10 ms; ionspray voltage, −4500 V; temperature, 600 °C; curtain gas, 45 psi; ion source gas 1, 60 psi; ion source gas 2, 60 psi. All data were analysed and processed using Analyst version 1.7.2 and SciexOS version 2.0 software packages. Concentrations were calculated based on the peak areas for the endogenous compounds relative to those determined for the internal standards.

### 4.5. Gene Expression Measurements by Quantitative Polymerase Chain Reaction (qPCR)

Total RNA was extracted from 100 mg of frozen ground tissue from the sample (S) leaf, using the Spectrum Plant Total RNA kit (Sigma-Aldrich, Auckland, New Zealand), following the supplier’s recommendations. All the 50 µL RNA extracts were then dried in a centrifugal evaporator (Refrigerated CentriVap Concentrator, Labconco, Kansas City, MO, USA) and re-suspended in a lower volume of nuclease-free water (15 µL in 2016 and 18 µL in 2018). DNAse treatment was carried out on 1 µg of RNA (for each sample) using the Quanta PerfeCTa DNA kit (DNAture Diagnostics & Research Ltd., Gisborne, New Zealand) according to the manufacturer’s instructions. First-strand cDNA was synthesised, with random priming, in a 20-μL reaction volume containing 1 μg of DNase-treated RNA, using the Quanta qScript cDNA Supermix kit (DNAture, Gisborne, New Zealand). Non-template controls included in each PCR plate were used to check the purity of the reagents.

A randomised subset of samples was tested by qPCR against seven-candidate kiwifruit reference genes (RG) identified in previous studies [59]. The geNorm v3.5 (Microsoft^®^ Excel) was then used to select the two most stably expressed RGs, which were *Actin* and *40s ribosomal protein* in 2016, and *GAPDH* and *PP2A* in 2018 (Supplementary Table S3). A gene expression normalisation factor was then calculated for the relative expression of each GoI using geNorm v3.5, based on the geometric mean of the two RG as described by Vandesompele, et al. [60].

Genes of Interest (GoIs) were chosen based on their involvement in induced kiwifruit resistance to scale insect or other kiwifruit pests and because they were reliable markers of different hormonal pathways [3,12,27,29,30,31,32,61,62] (Supplementary Table S3). *RD22* replaced *ABA1* as the ABA pathway marker in 2018. *PR5*, an SA-pathway marker induced by ASM treatment in kiwifruit [28], and *LOX2*, a commonly used JA-pathway marker [63], were also added to the 2018 qPCR screen.

The qPCR analyses were performed in triplicate on each of three biological replicates on a Corbett Rotor-Gene™ 6000 system (Corbett Life Science, Concorde, NSW, Australia). The 10-μL reactions contained 1 µL of a 10-fold dilution of the cDNA (for low-expressed samples, runs were repeated with 2 µL), 1 μM of each of forward and reverse primers and 5 μL of Light Cycler^®^ 480 SYBR Green 1 Master Mix (Roche Diagnostics GmbH, Mannheim, Germany, Product No. 04 887 352 001). The relative quantification thermal cycling conditions were: denaturation at 95 °C for 10 min, followed by 45 cycles of 10 s denaturation at 95 °C, 5 s annealing at a different optimised temperature between 55 and 60 °C for each primer set and 20 s extension at 72 °C. Inter-run variability was controlled by including a complete set of treatments on each plate with a separate run for each biological replicate (i.e., three runs/primer set, which were then averaged). Melting curve analysis (60–95 °C at 1 °C increments with 5 s between each step) was performed after the final qPCR cycle to validate amplicon specificity.

Apart from *Actin* and *PR1* [27] and *PP2A* [64], all other primers were designed in-house using Primer3 software (Whitehead Institute, Cambridge, MA, USA) and were synthesised by Invitrogen (Auckland, New Zealand) (Supplementary Table S3). Only primer pairs with efficiencies of 80% or greater were used in the experiments.

### 4.6. Gene Expression Measurements by NanoString

NanoString analysis was carried out only on ‘Hort16A’ and ‘Hayward’ tissue from the 2018 experiment at Grafton Clinical Genomics (the University of Auckland, Auckland, New Zealand). Total RNA from 100-mg samples of ground kiwifruit tissue was extracted, and the concentration and purity were assessed as described for qPCR analysis. Working aliquots of 50 ng/µL were prepared with nuclease-free water and stored at −80 °C.

The most stable RGs and the best differentially expressed GoIs from the qPCR experiment (Supplementary Table S3) were also used for NanoString. Additional GoIs (Supplementary Table S4) were selected based on a previous study on the kiwifruit molecular response to latania scale [3] to represent as many different defence response pathways and temporal stages of defence as possible. Oligomer design for the RG and GoI probes was carried out by NanoString Technologies Inc. (Seattle, WA, USA), and probes were synthesised by Integrated DNA Technologies Private Limited (IDT, Singapore).

Using a NanoString titration-24 kit and different RNA inputs, an overnight hybridisation of 18 h at 67 °C was carried out to determine the optimal RNA input (ng) for 150,000 total barcode counts as the optimum level as per NanoString recommendations [65]. The resulting optimal RNA input (60 ng for the 2018 ‘Hort16A’ and ‘Hayward’ tissue) was used in the full PlexSet run. For the PlexSet run, the 15-µL reaction volume in each well contained: 5 µL hybridisation buffer; 0.5 µL working probe A (0.6nM each of Probe As); 0.5 µL working probe B (3nM each of Probe Bs); 2 µL of the appropriate PlexSet (A-H); and 7 µL of sample RNA (8.6 ng/µL for the 2018 experiment). The plate was hybridised in the thermocycler at 67 °C for 19 h. The raw count data (number of hybridised target sequences for each gene) that were generated from PlexSet were analysed to ensure that quality control parameters (relating to successful image capture and reporter probe binding density) were met and were normalised against all the reference genes (Supplementary Table S4) using the nSolver™ software (version 4.0) provided by NanoString Technologies Inc. (Seattle, WA, USA). Normalised data were then statistically analysed, as described for qPCR results.

### 4.7. Statistical Analysis

Data for all the variables were transformed when necessary to meet the analysis assumptions of variance (ANOVA), i.e., Normal distribution and homogeneity of variances; means were then backtransformed for ease of interpretation in graphs and tables. Fisher’s least significant difference (LSD) was used post hoc for treatment means comparisons, at *p* ≤ 0.05. The analyses were conducted using Genstat 20th edition (VSN International). There were five biological replicates per treatment for all phytohormone and gene expression measurements.

## 5. Conclusions

‘Hort16A’ kiwifruit tolerance to latania scale is associated with increased responsiveness to elicitors of the SA pathway—whether this is the latania scale insect itself or the use of an elicitor such as ASM, which is a SA analogue. Whilst gene expression proved to be a more reliable resistance marker than phytohormones, the susceptible ‘Hayward’ cultivar had greater concentrations than ‘Hort16A’ of inert storage forms of both SA and JA across all treatments. Antagonistic crosstalk between the SA and JA pathways was not evident in that use of a JA pathway elicitor, MeJA, had no notable effect on latania scale growth and survival or gene expression in either cultivar and led to an increased concentration of inert storage forms of JA in ‘Hayward’ versus ‘Hort16A’. This information contributes to the selection of tolerant cultivars and the effective use of elicitors for control of latania scale in kiwifruit.

## Figures and Tables

**Table 1:** Treatments applied to ‘Hort16A’ and ‘Hayward’ kiwifruit plants.

Treatment Code	Treatment Details
Control	Control—plants left untreated.
Wounding	Sample leaf mechanically wounded one week after the start of the trial.
Latania	Water + 0.025% *v*/*v* DuWett^®^ sprayed on day 1, 200+ latania scale crawlers applied to the sample leaf, one week later.
ASM	Actigard™ (ASM, 0.2 g/L) applied to all leaves on day 1.
ASM + Latania	Same treatment as above, plus latania scale application one week later.
MeJA	Methyl jasmonate (0.05% *v*/*v*) + 0.025% (*v*/*v*) DuWett^®^ applied to all leaves on day 1.
MeJA + Latania	Same treatment as above, plus latania scale application one week later.

## Data Availability

Data are contained within the article or Supplementary Materials.

## Supplementary Materials

The following are available online at https://www.mdpi.com/article/10.3390/plants11010010/s1, Protocol S1. Extraction of leaf samples for liquid chromatography/mass spectrophotometry (LC/MS) in 2016. Protocol S2. Extraction of leaf samples for LC/MS in 2018, Table S1: Compounds used in liquid chromatography/mass spectrophotometry (LC/MS), Table S2: Multiple reaction monitoring (MRM) transitions used for plant hormone analysis, Optimised Q1 (precursor ion) and Q3 (product ion) transitions, retention time (RT), declustering potential (DP), entrance potential (EP), collision energy (CE) and collision cell exit potential (CXP) for each of the phytohormones analysed. The internal standard (IS) used for quantitation for each phytohormone is also given. SAG = salicylic acid O-β-glucoside; 12-OH-JA = 12-hydroxy jasmonic acid; SA = salicylic acid; ABA = abscisic acid; JA = jasmonic acid; DH-JA = 9,10-dihydrojasmonic acid; JA-Ile = jasmonoyl-isoleucine; OPC-4 = (+/−)-4-(3-oxo-2-(pent-2-enyl)cyclopentyl) butanoic acid; and cis-OPDA = cis-(+)-12-oxo-phytodienoic acid. Table S3: Identification of reference genes (RGs) and genes of interest (GoI) and their primer sequences used for ‘Hort16A’ and ‘Hayward’ kiwifruit leaf defence gene expression analysis by a quantitative polymerase chain reaction in 2016 and 2018, Table S4: Identification of reference genes (RGs) and genes of interest (GoI) and their primer sequences used defence gene expression analysis by nCounter™ PlexSet™ NanoString on ‘Hort16A’ and ‘Hayward’ kiwifruit (*Actinidia chinensis*) leaf tissue in 2018.
