# Supplementary material for: Defence Responses Associated with Elicitor-Induced, Cultivar-Associated Resistance to Latania Scale in Kiwifruit
Source: Plants (Basel). 2021 Dec 21;11(1):10. doi: 10.3390/plants11010010 (PMC8747134; doi:10.3390/plants11010010)
Supplement: Supplementary file 1 [file plants-11-00010-s001.zip › 21590 - Kirstin Wurms Scale 1 and 2 manuscript supplementary data v5.pdf]

Protocol S1. Extraction of leaf samples for liquid chromatography/mass spectrophotometry (LC/MS) in 2016.

For each sample, 100 mg frozen ground tissue (FW) was weighed into a 2-mL Eppendorf tube on dry ice and extracted with 1 mL of chilled (4°C) 80:20 acetonitrile:water to which internal standards had been added (2.5 ng [$^2H_4$] SA, 12.5 ng [$^2H_5$] JA, and 6.25 ng [$^2H_6$] ABA). The samples were shaken for 30 min at 4°C on a flat-bed orbital shaker, extracted overnight at −20°C and then centrifuged at 13,000 g for 10 min at 4°C. An 800-μL aliquot of each sample was transferred to a 96-well deep plate and evaporated to dryness in a refrigerated CentriVap vacuum concentrator at −4°C (Labconco, Kansas City, MO, USA). Samples were reconstituted in 200 μL of chilled (4°C) 80:20 acetonitrile:water and then made basic with the addition of 1.8 mL of 0.67% ammonium hydroxide (aq) prior to SPE clean-up on a SOLA SAX 96-well plate (10 mg/2 mL; Thermo Scientific, CA, USA). Plates were activated using 2x 1.5 mL acetonitrile and equilibrated using 2x 1.5 mL 0.67% ammonium hydroxide (aq). After equilibration, samples were loaded and washed with 1.5 mL of 0.67% ammonium hydroxide (aq) followed by 1.3 mL of methanol. Acidic plant hormones were eluted with 600 μL 3.77% formic acid in acetonitrile and evaporated to dryness in a refrigerated centrivap vacuum concentrator at −4°C. Samples were reconstituted in 200 μL of 10:90 acetonitrile:water for analysis by LC/MS.

Protocol S2. Extraction of leaf samples for LC/MS in 2018.

The samples, 100 mg frozen ground tissue (FW), were weighed into 1.5-mL Eppendorf tubes on dry ice, and to each was added 1 mL chilled (4°C) extraction solvent (acetonitrile + 0.01% TFA), labelled internal standard mix (2.5 ng [$^2H_4$] SA, 25 ng [$^2H_5$] JA, 6.4 ng [$^2H_6$] ABA, 0.6 ng [$^2H_{10}$] JA-ile (2018 season only)) and 0.8 g stainless steel beads 0.9–2 mm (Next Advance Inc., NY, USA). Samples were worked up as described in Bulley et al. [1] and reconstituted in 200 μL of 10:90 acetonitrile:water for analysis by LC/MS.

Table S1. Compounds used in liquid chromatography/mass spectrophotometry (LC/MS)

Compound Name	Abbreviation	Source
Radio-labelled salicylic acid O-β-glucoside	[$^2H_4$] SAG	synthesised utilising [$^2H_4$][1] MeSA in place of MeSA [2]
Salicylic acid O-β-glucoside	SAG	synthesised using published methodology [2][2]
12-hydroxy jasmonic acid	12-OH-JA	Ol-chemim Ltd (Olomouc, Czech Republic)
Radio-labelled salicylic acid	[$^2H_4$] SA	CDN Isotopes (Pointe-Claire, Quebec, Canada)
Salicylic acid	SA	Sigma Aldrich (Auckland, New Zealand)
Radio-labelled abscisic acid	[$^2H_6$] ABA	Toronto Research Chemicals (Toronto, Ontario, Canada)
Abscisic acid	ABA	Sigma Aldrich (Auckland, New Zealand)
Radio-labelled jasmonic acid	[$^2H_5$] JA	CDN Isotopes (Pointe-Claire, Quebec, Canada)
Jasmonic acid	JA	Sigma Aldrich (Auckland, New Zealand)
9,10-dihydrojasmonic acid	DH-JA	Ol-chemim Ltd (Olomouc, Czech Republic)
Radio-labelled jasmonoyl-isoleucine	[$^2H_{10}$] JA-Ile	synthesised utilising [$^2H_{10}$] L-isoleucine in place of isoleucine [3][1]
Jasmonoyl-isoleucine	JA-Ile	Ol-chemim Ltd (Olomouc, Czech Republic)
(+/-)-4-(3-oxo-2-(pent-2-enyl)cyclopentyl) butanoic acid	OPC-4	Ol-chemim Ltd (Olomouc, Czech Republic)
cis-(+)-12-oxo-phytodienoic acid	cis-OPDA	Ol-chemim Ltd (Olomouc, Czech Republic)

[1]>99% pure by LC/MS.

[2]>99% pure by ^1H- and ^{13}C-NMR.

Table S2. Multiple reaction monitoring (MRM) transitions used for plant hormone analysis. Optimised Q1 (precursor ion) and Q3 (product ion) transitions, retention time (RT), declustering potential (DP), entrance potential (EP), collision energy (CE) and collision cell exit potential (CXP) for each of the phytohormones analyzed. The internal standard (IS) used for quantitation for each phytohormone is also given. SAG = salicylic acid O-β-glucoside; 12-OH-JA = 12-hydroxy jasmonic acid; SA = salicylic acid; ABA = abscisic acid; JA = jasmonic acid; DH-JA = 9,10-dihydrojasmonic acid; JA-Ile = jasmonoyl-isoleucine; OPC-4 = (+/–)-4-(3-oxo-2-(pent-2-enyl)cyclopentyl) butanoic acid; and cis-OPDA = cis-(+)-12-oxo-phytodienoic acid.

Compound	Q1	Q3	RT (min)	DP	EP	CE	CXP	IS
[$^2H_4$] SAG	303.00	96.90	4.53	-50	-10.0	-56.2	-8.6	-
SAG	299.30	136.80	4.54	-60	-10.0	-20.0	-12.0	[$^2H_4$] SAG
12-OH-JA	225.10	58.90	4.78	-60	-10.0	-20.0	-7.9	[$^2H_5$] JA
[$^2H_4$] SA	141.10	97.00	4.98	-40	-7.8	-40.0	-9.6	-
SA	136.90	93.00	4.99	-40	-11.0	-25.0	-7.0	[$^2H_4$] SA
[$^2H_6$] ABA	269.00	159.00	5.28	-25	-10.0	-17.0	-15.0	-
ABA	263.10	153.00	5.28	-60	-5.0	-15.0	-8.0	[$^2H_6$] ABA
[$^2H_5$] JA	214.00	62.10	5.51	-60	-6.0	-24.0	-8.6	[$^2H_5$] JA
JA	209.10	59.10	5.52	-60	-6.0	-24.0	-8.6	[$^2H_5$] JA
DH-JA	211.00	59.00	5.71	-100	-2.9	-20.0	-5.0	[$^2H_5$] JA
[$^2H_{10}$] JA-Ile	332.20	139.90	5.83	-60	-10.0	-26.9	-12.0	-
JA-Ile	321.90	129.80	5.84	-25	-10.0	-32.0	-15.0	[$^2H_{10}$] JA-Ile
OPC-4	237.20	58.80	5.84	-25	-10.0	-23.0	-15.0	[$^2H_5$] JA
cis-OPDA	291.00	165.00	6.40	-48	-10.0	-26.0	-3.0	[$^2H_5$] JA

Table S3. Identification of reference genes (RGs) and genes of interest (GoI) and their primer sequences used for 'Hort16A' and 'Hayward' kiwifruit leaf defence gene expression analysis by quantitative polymerase chain reaction in 2016 and 2018.

Gene name (Gene ID used in Figures).	RG or GoI	Identification (NCBI entry[1]/Acc #[2]/Achn number[3])	Forward primer (5′-3′)	Reverse primer (5′-3′)
40s ribosomal protein	RG	FG498176/Acc23370.1	GCAAAGGGATGTGAGGTGAT	CCCCCTGTCTCAGAAGAACA
Glyceraldehyde 3-phosphate dehydrogenase (*GAPDH*)	RG	FG499278/Acc18050.1	GCAGTCAACGATCCCTTCAT	GACGCCGAAAACTGTGACTT
Protein phosphatase 2A (*PP2A*)	RG	FG522516/Acc33246.1	GCAGCACATAATTCCACAGG	TTTCTGAGCCCATAACAGGAG
Actin	RG	EF063572/Acc05529.1	CACCCTGTGCTGCTTACAGA	GAGAGAGAACGGCCTGAATG
**<u>SA associated genes</u>**				
Pathogenesis-related protein family 1 (*PR1*)	GoI	FG499230/Acc06864.1	GCCCCCGGTAAGGTTTGT	CGAACCAAGACCCACTATTGC
Glucan endo-1,3-β-glucosidase (*β-1,3-glucosidase*)	GoI	FG455092/Acc03929.1	TTGGTTCAACATGTCAAAGGAG	TAGGCTGCTTGTTGGGAAAG
Pathogenesis-related protein family 5 (*PR5*)	GoI	AJ871175/Acc28854.1	CGATGGAATTTAGCCCTACG	ATTTCCGGAGTTGCAACAGT
NPR1	GoI	Acc00932.1	AGTTCCCATTGTCCGTCATC	AAGAAACGCTTCCCCAATTC
**<u>JA associated genes</u>**				
Lipoxygenase (*LOX2*)	GoI	Acc32487.1/Achn123621	CAGAGCTTGATCCCAAGACA	CAGGATGGCTCCAATTTCAC
APETALA2 Ethylene responsive factor 2 (*AP2 ERF2*)	GoI	Acc20680.1/Achn033321	ATGAGTCCAATGGCCAAAAC	GATTTCTGCCGCATATTTCC
Jasmonoyl-L-amino acid synthetase (*JAR1*)	GoI	Acc09200.1	TTCTACATTTGCCCACAGCA	TTTGGCTTGAGCAGTTTTGA
**<u>ABA associated genes</u>**				
Response to desiccation 22 (*RD22*)	GoI	Acc29141.1	GACCTCAAAAAGGGCACAAA	AGTCGGGCTTCACAGAAAAG

Abscisic acid deficient 1 (*ABA1*)	GoI	Acc10322.1/Achn013171	GTAGATGGTCCCGCTGGTAA	TTTCCCCACGTAAAAATTGG

[1]NCBI = National Centre for Biotechnology Information.
[2]Acc. numbers come from the manual annotation of the Red5 kiwifruit whole Genome Shotgun project [4] that has been deposited at DDBJ/ENA/GenBank.
[3]Achn numbers identify *A. chinensis* var. *chinensis* 'Hongyang' genome sequences from the project described by Huang et al. [5]. The sequences are found at http://bioinfo.bti.cornell.edu/cgi-bin/kiwi/home.cgi.

Table S4. Identification of reference genes (RGs) and genes of interest (GoI) and their primer sequences used defence gene expression analysis by nCounter™ PlexSet™ NanoString on 'Hort16A' and 'Hayward' kiwifruit (*Actinidia chinensis*) leaf tissue in 2018.

Gene name (Gene ID used in Figures)	RG or GoI	Identification (NCBI entry[1]/Acc #[2]/Achn number[3])	Primer A	Primer B
40s ribosomal protein	RG	FG498176/<br>Acc23370.1	AAACGCCATAGCAGGCCCTGCG<br>AACAGCGAGGCCACCAAGGAGC<br>TCATCCTCTTCTTTTCTTGGTGTT<br>GAGAAGATGCTC	CGAAAGCCATGACCTCCGATCA<br>CTCACTAACAATCACCTCACATC<br>CCTTTGCCCCGCTCTCCATAACA<br>AATCTCA
Glyceraldehyde 3-phosphate dehydrogenase (GAPDH)	RG	FG499278/<br>Acc18050.1	AATCCCAGCCTTGGCATCAAAG<br>ATGCTCGATCTGCTGTCACCAAC<br>AAAGTCTGTTGAGATTATTGAGC<br>TTCATCATGACCAGAAG	CGAAAGCCATGACCTCCGATCA<br>CTCCACTCGTTGTCATACCAAGA<br>CACCAGTTTCACGAACAAGTCGT<br>TCAAAGC
Protein phosphatase 2A (*PP2A*)	RG	FG522516/<br>Acc33246.1	TAATCATGTCCAATACCTGTGGA<br>ATTATGTGCTGCATTGCCCATTC<br>TGGACAAAGACGCCTATCTTCCA<br>GTTTGATCGGGAAACT	CGAAAGCCATGACCTCCGATCA<br>CTCAAGAGAGATCGAGTGTAGT<br>ATGGTCATACGGTACAGATAAT<br>GTGGGTTGC
Actin	RG	EF063572/<br>Acc05529.1	GTTGAACCACCACTGAGCACAA<br>TGTTGCCATATAGATCTTTCCTG<br>ATATCCGAACCTAACTCCTCGCT<br>ACATTCCTATTGTTTTC	CGAAAGCCATGACCTCCGATCA<br>CTCCAAGAGCAGTAATTTCCTTA<br>CTCATGCGGTCTGCGATACCAGG<br>GAACATA
Elongation Factor (*EF*)	RG	FG526520/<br>Acc31629.1	TTCCCCAACTTTAATCGTCCCTTGT<br>TCAACACGACCAGTGGCAACAGTT<br>CCCTCAAGACCTAAGCGACAGCGT<br>GACCTTGTTTCA	CGAAAGCCATGACCTCCGATCACT<br>CACCGTAGATTTCATGGAGCACC<br>CTGCATCAGCCCTAAAATCTCAAC<br>ATC
**<u>SA associated genes</u>**				
Pathogenesis-related protein family 1 (*PR1*)	GoI	FG499230/<br>Acc06864.1	CTTCTCTGCAACCCATAGGTTCA<br>CTGCAGCGGTGCCAGTAAAAGA<br>TCCACCCAATTTGGTTTTACTCCC<br>CTCGATTATGCGGAGT	CGAAAGCCATGACCTCCGATCA<br>CTCTGCAAGCATTCTCCCCCGAC<br>ACAAGAGTTAGAGTTGATATCAT<br>AGTTCGG
Glucan endo-1,3-β-glucosidase (*β-1,3-glucosidase*)	GoI	FG455092/<br>Acc03929.1	ACATACTCTATTTTTTGCTAGTGC<br>CCTCTTTATGAGGGAAATCACAA<br>GCACAACAGCCACTTTTTTTCCA<br>AATTTTGCAAGAGCC	CGAAAGCCATGACCTCCGATCA<br>CTCTATTCCTTTATTAGATATTTT<br>GTCTGTCTTCATAGGAGCAATCT<br>CTCGGT
Pathogenesis-related protein family 5 (*PR5*)	GoI	AJ871175/<br>Acc28854.1	CCTGGAACGGCAGCGGCCCAAA<br>CGGTGAAAGGGCAGTTGTTTATG<br>ATATTCCTGGAGTTTATGTATTG<br>CCAACGAGTTTGTCTTT	CGAAAGCCATGACCTCCGATCA<br>CTCCAGGATTGATGATCCAATTC<br>TGGCCACGGTCAAGGCGTTTGCC<br>ACCA
WRKY33	GoI	FG468929	CGAAAGCCATGACCTCCGATCA<br>CTCAGATTTCTGTGGAGAAGCTT<br>TGAATGGGTGCAAATTCTGATTT<br>CCCTCCA	GGGTTAAACGACGAGAATTGAG<br>TTTCCTCTGATGGTTTGTTCATGT<br>TCCACACCGTGTGGACGGCAAC<br>TCAGAGATAACGCATAT
WRKY75	GoI	FG474406	TCCAATTGAGAAGTCCACAGAC<br>AACTTTGAGCACATCTTGACTCA<br>GATGCCCTGGAGTTTATGTATTG<br>CCAACGAGTTTGTCTTT	CGAAAGCCATGACCTCCGATCA<br>CTCCCTAAAGATGAAGGAGTGG<br>TGGTGACAACTTATGAAGGCAT<br>GCACTCCCA

Glutathione S-transferase (*Glut-S-transferase*)	GoI	FG460267	ATCGATCTTGTTCAGCTCTTCAA<br>TCCATGCTGCTAGTTTAGGCCGC<br>AGATAAGGTTGTTATTGTGGAGG<br>ATGTTACTACA	CGAAAGCCATGACCTCCGATCA<br>CTCCTGTATTGTTTGACCAATGC<br>TTCAGGGTCGCATTTTGTCTGCG<br>TGTAAGC
Hypersensitive response/ salicylic acid signalling (*HS SA sig*)	GoI	FG431814	ACATATACTGCATAGTTTCTCTA<br>GCGGTTCATCTCCAGCTTCTGCC<br>TGAGGCTGTTAAAGCTGTAGCA<br>ACTCTTCCACGA	CGAAAGCCATGACCTCCGATCA<br>CTCTACGGGTAGGTGAGAAGGT<br>TTACCAATGACAAGTTCACTGCC<br>TCCCTTTC
Pathogenesis-related protein family 2 (*PR2 Primer3*)	GoI	FG480859	TCAAACATGGCAAACACATAAG<br>TTTCTATCGGTCCACCGGACCTT<br>CTAGGCTAGGACGCAAATCACT<br>TGAAGAAGTGAAAGCGAG	CGAAAGCCATGACCTCCGATCA<br>CTCGGAGAAAGAGACCCCAATG<br>CTTCTCAAATTCTGGGTCTTTTTG<br>ATTCTCA
Pathogenesis-related protein family 5 (*PR5 PrimerF*)	GoI	FG417283/ Acc28852.1	ATTGCCCGTGATATTGACAGAGT<br>ACGATATTGGCTGACACTTGCCT<br>GACACCACGCGATGACGTTCGT<br>CAAGAGTCGCATAATCT	CGAAAGCCATGACCTCCGATCA<br>CTCAAAACCGTGCACAGGTTATT<br>GCATTGCCTGGGTTTGTAACGGG<br>TTTGGGC
Endochitinase (*Endochit PrimerG*)	GoI	FG512537/ Acc24397.1	AAAAGCATCATAGGTATAAAAT<br>CCCTTCCCCTCGCAAGCGGCATC<br>GTTCCCATTTGGAATGATGTGTA<br>CTGGGAATAAGACGACG	CGAAAGCCATGACCTCCGATCA<br>CTCGCAGCATCACCGGTGTTTCC<br>AAAAGCCCCAAAAGACTTAGCA<br>GCAGAAAC
**<u>JA associated genes</u>**				
Cytochrome oxidase subunit 1 (*COI1*)	GoI	FG460354	CGTTGTCTTGCTCACTTTTCTGTC<br>TTCTTCCATAAGATCTGAACCGC<br>AAACTTCCTTCCTGTGTTCCAGC<br>TACAAACTTAGAAAC	CGAAAGCCATGACCTCCGATCA<br>CTCTAGTCAATCACGCGCTGGAA<br>CACCAAATCCGGCATTCCGTTTC<br>CATCACT
Lipoxygenase (*LOX2*)	GoI	Acc32487.1/ Achn123621	TCTTCTCAGAATCATCAGACTTG<br>GAAGCAACCCAAGAGTTGCAAT<br>TCACACATAAAATTGGTTTTGCC<br>TTTCAGCAATTCAACTT	CGAAAGCCATGACCTCCGATCA<br>CTCCTCTCTTAGCCTCTTTAACCC<br>ATTTGGTGTCTTGTTGGTGAAGA<br>AAACTC
Plant defensin gene 1.2 (*PDF1.2*)	GoI	Acc11160.1	TCTCGCTCCGGCGCTGGCATAAC<br>TTTGCTGTTGCGAATTCAGAGTT<br>AGCTCTTTCGTTGGGACGCTTGA<br>AGCGCAAGTAGAAAAC	CGAAAGCCATGACCTCCGATCA<br>CTCACTGTCTGTCGCAGTTATCA<br>GTCGAGCCACAGGGCCCGGACC<br>ATG
**<u>Phenyl propanoid pathway genes</u>**				
Phenylalanine ammonia lyase (*PAL*)	GoI	FG484623/ Acc32206.1 /Achn060261	CATCTTAGTCGATGAGCGGATGA<br>CTTCGATTAGCGGGCCCAACCACC<br>TGGGCTGGTCAAGACTTGCATGA<br>GGACCCGCAAATTCCT	CGAAAGCCATGACCTCCGATCA<br>TCGAGACATCGATCAATGGATT<br>ATCGTTCACCGAGTTGATCTCCC<br>GTTCGAT
Chalcone synthase (*CHS*)	GoI	FG528287	AAGAGAGAGTTCCAGTCTGAGA<br>TGCCCAAGGGCTTGAAAGCCTCT<br>ACTAGCCAGCAGACCTGCAATA<br>TCAAAGTTATAAGCGCGT	CGAAAGCCATGACCTCCGATCA<br>CTCTCAGTTCGACTTGGTCTAAA<br>ATAACAGGCCCACCAGGGTGAG<br>CGATCCAA
4-Coumarate-coenzyme A ligase (*4CL*)	GoI	FG515208	AACTTCACCAGCAACTTCATCTT<br>TTTGCGGGACAACAGCAGCGTC<br>GGCAACCTGCCAATGCACTCGA<br>TCTTGTCATTTTTTTGCG	CGAAAGCCATGACCTCCGATCA<br>CTCGCTTCTTCTGTAAGTTCAAA<br>CCCATTCGACCGAACCACAAAT<br>GCTACAGG

Cinnamoyl-coenzyme A reductase (*CCR*)	GoI	FG397115	TCCAGCAGATCTACCTTGAAGAG TTTCAGATTCTCGGATGCTTTATC AAGCAAACTGGAGAGAGAAGTG AAGACGATTTAACCCA	CGAAAGCCATGACCTCCGATCAC TCCATGGAACACTCCGTCGCATC CTGATATGGGTGCAGAAAGAGA ATGGTAA
**Defence hub gene**				
TCP14 hub protein (*TCP14 hub*)	GoI	FG496474	GACCGTCGCTTCCGTCCATGATC ACATGAGAAATACTCTCTCTCTCT CTCCGATTGCTGCATTCCGCTCAA CGCTTGAGGAAGTA	CGAAAGCCATGACCTCCGATCAC TCCTTCCTCTTTCTTCTCGAGCAG TTGGAATGGGAAATTCGGGCGGT GCA

[1]NCBI = National Centre for Biotechnology Information
[2]Acc. numbers come from the manual annotation of the Red5 kiwifruit whole Genome Shotgun project [4] that has been deposited at DDBJ/ENA/GenBank
[3]Achn numbers identify *A. chinensis* var. *chinensis* 'Hongyang' genome sequences from the project described by Huang et al. [5]. The sequences are found at http://bioinfo.bti.cornell.edu/cgi-bin/kiwi/home.cgi.

References

1. Bulley, S.M.; Cooney, J.M.; Laing, W. Elevating ascorbate in *Arabidopsis* stimulates the production of abscisic acid, phseic acid, and to a lesser extent auxin (IAA) and jasmonares, resulting in increased expression of DHAR1 and multiple transcription factors associated with abiotic stress tolerance. *International Journal of Molecular Scienes* **2021**, *22*, 6743, doi:https://doi.org/10.3990/ijms22136743.
2. Grynkiewicz, G.; Achmatowicz, O.; Hennig, J.; Indulski, J.; Klessig, D.F. Synthesis and characterization of the salicylic-acid beta-d-glucopyranoside. *Pol. J. Chem.* **1993**, *67*, 1251-1254.
3. Kramell, R.; Schmidt, J.; Schneider, G.; Sembdner, G.; Schreiber, K. Synthesis of n-(jasmonoyl)amino acid conjugates. *Tetrahedron* **1988**, *44*, 5791-5807, doi:10.1016/s0040-4020(01)81437-x.
4. Pilkington, S.M.; Crowhurst, R.; Hilario, E.; Nardozza, S.; Fraser, L.; Peng, Y.Y.; Gunaseelan, K.; Simpson, R.; Tahir, J.; Deroles, S.C.*, et al.* A manually annotated *Actinidia chinensis* var. chinensis (kiwifruit) genome highlights the challenges associated with draft genomes and gene prediction in plants. *BMC Genomics* **2018**, *19*, 19, doi:10.1186/s12864-018-4656-3.
5. Huang, S.X.; Ding, J.; Deng, D.J.; Tang, W.; Sun, H.H.; Liu, D.Y.; Zhang, L.; Niu, X.L.; Zhang, X.; Meng, M.*, et al.* Draft genome of the kiwifruit *Actinidia chinensis*. *Nature Communications* **2013**, *4*, 9, doi:10.1038/ncomms3640.
